# Stereotactic Body Radiotherapy for Localized Ureter Transitional Cell Carcinoma: Three Case Reports

**DOI:** 10.1155/2015/519897

**Published:** 2015-09-09

**Authors:** Yoshiyasu Maehata, Kengo Kuriyama, Shinichi Aoki, Masayuki Araya, Kan Marino, Hiroshi Onishi

**Affiliations:** ^1^Department of Radiation Oncology, Kofu Municipal Hospital, 366 Masutsubo, Kofu, Yamanashi 4000832, Japan; ^2^Department of Radiation Oncology and Radiology, University of Yamanashi School of Medicine, 1110 Shimokato, Chuo, Yamanashi 4093898, Japan

## Abstract

The gold standard management for ureter transitional cell carcinoma (UTCC) is radical nephroureterectomy with excision of the bladder cuff. However, some patients cannot undergo this procedure for several reasons. In the case reports described herein, we performed stereotactic body radiotherapy (SBRT) on three patients with inoperable or surgery-rejected localized UTCC. Two out of the three patients did not develop local recurrence or distant metastasis during the observation period. However, recurrence was detected in the bladder of one patient 22 months after the treatment. No acute or late adverse events occurred in any of the three patients. SBRT may become one of the treatment options for inoperable or surgery-rejected UTCC patients.

## 1. Introduction

Ureter transitional cell carcinoma (UTCC) is rare, accounting for only 1–3% of urinary transitional cell carcinoma cases [1]. The standard treatment for UTCC is surgical resection. Since TCC may develop synchronously or metachronously at different urinary tract sites in a multifocal manner, radical nephroureterectomy (RNU) with excision of the bladder cuff is generally recommended [2]. However, some patients do not undergo RNU due to comorbidities or refusal of the procedure. We herein presented three patients with localized UTCC who were treated with stereotactic body radiotherapy (SBRT).

## 2. Case Presentation

### 2.1. Case 1

**Figure 1:**
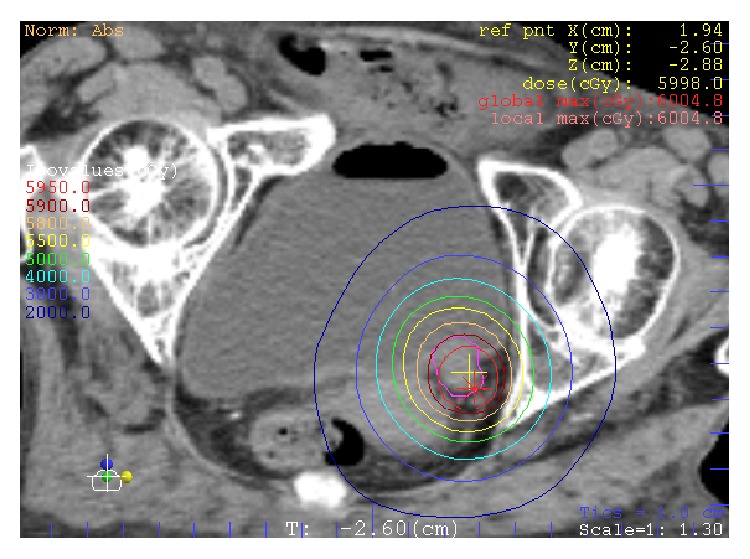
SBRT plan isodose line of case 1.

An 87-year-old woman with rheumatism presented with gross hematuria and dysuria. A cystoscopic examination showed a papillary tumor at the left ureteral orifice protuberating into the bladder. A histological examination confirmed transitional cell carcinoma grade 2. A CT scan revealed the mass at the distal ends of the left ureter with no lymphatic or distant metastasis. Therefore, this patient was diagnosed with ureter cancer T2N0M0. Radical nephroureterectomy was considered to be nonadaptive because of comorbidities of chronic kidney disease (CKD) and rheumatism; therefore, she was referred to our department. SBRT was performed by coplanar dynamic conformal radiotherapy with a linear accelerator (LINAC) (EXL-15DP, Mitsubishi Electric, Tokyo, Japan). A total dose of 60 Gy was delivered in 10 fractions at the center of planning target volume (PTV) using 10 MV photons (Figure 1). No acute radiation-related adverse events were observed during the treatment. The size of the tumor had decreased 3 months after the treatment, and there was no evidence of local recurrence or distant metastasis at 12 months on CT scans. The patient died from the exacerbation of rheumatism 33 months after the radiation treatment.

### 2.2. Case 2

**Figure 2:**
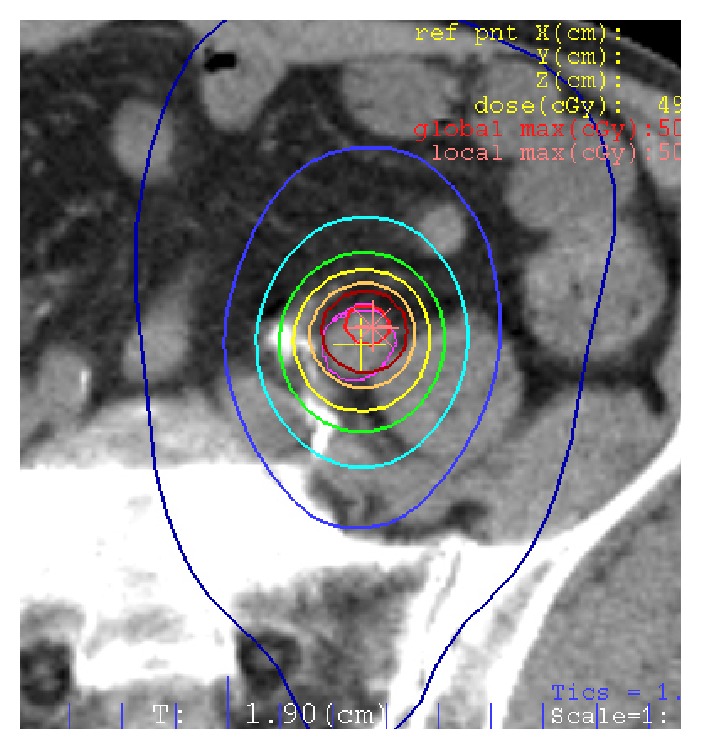
SBRT plan isodose line of case 2.

An 87-year-old man presented with occult hematuria. A cystoscopic examination showed no abnormal findings. The result of urine cytology was class V, transitional cell carcinoma. A CT scan showed a small tumor at the distal one-third of the left ureter with dilation of the upper ureter and ipsilateral renal pelvis. There was no evidence of lymphatic or distant metastasis. Therefore, the patient was diagnosed with ureter cancer T2N0M0. The patient refused surgery and was referred to our department. SBRT was performed by noncoplanar dynamic arch radiotherapy with a LINAC. A total dose of 50 Gy was delivered in 10 fractions at the center of PTV using 10 MV photons (Figure 2). No acute radiation-related adverse events were observed during the treatment. The size of the tumor had decreased 3 months after the treatment, and dilation of the ureter and renal pelvis improved. Although the renal pelvis was slightly redilated 9 months after the treatment, there was no evidence of tumor regrowth on a CT scan 12 months after the treatment. The patient died of severe bacterial pneumonia 13 months after the radiation treatment.

### 2.3. Case 3

**Figure 3:**
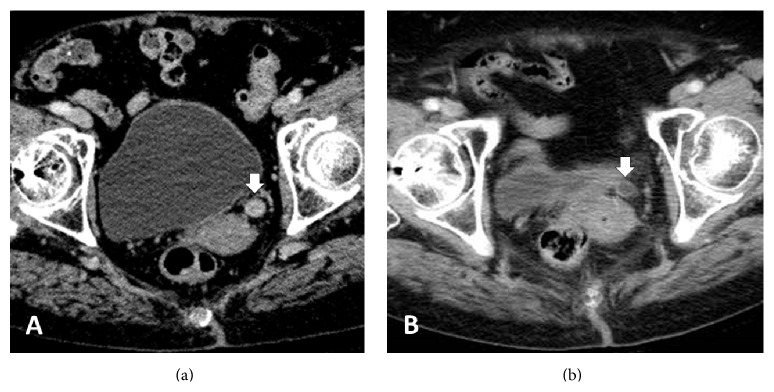
Contrast-enhanced CT scan of the patient. (a) A CT scan showed the tumor as enhanced thickness of the wall at the distal ends of the left ureter before the treatment (see arrow). (b) The enhanced thickness of the ureteral wall improved 3 months after SBRT.

An 85-year-old woman presented with gross hematuria. A cystoscopic examination revealed a tumor in the left ureter. A histological examination confirmed transitional cell carcinoma grade 3. A CT scan showed the tumor at the distal ends of the left ureter with no lymphatic or distant metastasis (Figure 3(a)). Therefore, the patient was diagnosed with ureter cancer T2N0M0. The patient refused surgery and was referred to our department. SBRT was performed by coplanar three-dimensional conformal radiotherapy with a LINAC. A total dose of 60 Gy was delivered in 10 fractions at the center of PTV using 10 MV photons. No acute radiation-related adverse events were observed during the treatment. The size of the tumor had decreased 3 months after the treatment (Figure 3(b)). A cystoscopic examination revealed papillary tumors on the bladder neck 22 months after the treatment. The tumor was removed with transurethral resection of bladder tumor (TURBT), and the recurrence of UTCC was detected in the bladder. Two months later, tumors reappeared on the bladder neck. A CT scan indicated a bladder tumor with no evidence of local recurrence in the ureter or distant metastases. There were no indications for surgical resection, and the patient underwent best supportive care. The patient died from exacerbation of the recurrent bladder tumor 38 months after the radiation treatment.

## 3. Discussion

The standard treatment of UTCC is surgical resection. In the present study, summarized in Table 1, surgical resection was not suitable for any of the three patients due to comorbidities or refusal to undergo the procedure. There is currently no established strategy for these patients. The role of radiotherapy for ureter cancer is generally adjuvant treatment as postoperative radiotherapy (PORT). PORT for high stage transitional cell carcinoma of the renal pelvis or ureter is known to reduce local failure risk after surgery alone [3]. However, to the best of our knowledge, there are no reports of radiotherapy alone for ureter cancer. In the case of radiotherapy for genitourinary cancer, the existence of highly radiosensitive organs such as the small intestine and kidneys is important issue. In the case of bladder cancer, the small intestine exists in the head side of the bladder, and efficient dose for curative radiotherapy often can be prescribed by means of filling bladder. For this reason, there are not a few reports of curative radiotherapy for bladder cancer, even in very elderly patients with comorbidities [4]. However, in the case of UTCC, the small intestine exists closely around the ureter attached with kidney. Thus, the dose of conventional radiotherapy for UTCC needs to be restrained, and sufficient effects cannot be expected. SBRT is a highly precise and multidirectional radiation technique that heightens local control and lessens adverse effects. SBRT administers higher radiation doses at the target with lower doses to normal tissue around the target than conventional radiotherapy. SBRT is often used for isolated tumors in the lung, liver, and spinal column. Onishi et al. examined 245 patients with early stage lung cancer treated with SBRT in 13 Japanese institutions and reported a local recurrence rate of just 8.1% in cases prescribed more than 100 Gy on BED_10_ [5]. Based on the above findings, we selected SBRT for the purpose of irradiating high doses to target lesions in the ureter while suppressing exposure doses to the small intestine and kidneys.

In the present study, the prescribed doses differed between cases. In cases 1 and 3, the tumor was localized at the distal end or orifice of the ureter, and 60 Gy was delivered in 10 fractions, which was equal to 96 Gy on a biological effective dose of 10 (BED_10_). In contrast, in case 2, the tumor was localized at the distal one-third of the ureter, and the small intestine was in close proximity to the tumor. Therefore, the tumor was treated with 50 Gy in 10 fractions, equal to 75 Gy on BED_10_, in order to suppress the exposure dose to the small intestine. However, there were no decisive differences of local control in all three cases.

There are various techniques of SBRT in sight of irradiation and image guidance. The most commonly used one is a linear accelerator (LINAC), and we performed SBRT with a CT-LINAC system in all three cases. Incidentally, SBRT with dedicated machines, including cyberknife, is also often performed especially for localized prostate cancer in genitourinary organs [6].

The sizes of the tumors in all three cases had decreased within 3 months of the treatment. Furthermore, there was no evidence of recurrence in the observation period spanning between 13 and 24 months. Therefore, SBRT was considered sufficiently effective for local control. Cases 1 and 2 showed no evidence of local recurrence, lymph node relapse, or distant metastasis in the observation period (12 and 13 months, resp.). However, bladder relapse was detected in case 3 22 months after the treatment. Hall et al. examined 252 patients treated surgically for upper urinary tract TCC and reported 12 months as the median time of disease relapse in 67 patients (27%) [7]. The above findings indicate that SBRT prolonged survival times in all three cases. Ureter carcinoma often causes recurrence at different urinary tract sites in a multifocal manner, and RNU with excision of the bladder cuff has been recommended as a standard treatment. Bladder recurrence occurred repeatedly in case 3. In contrast, segmental ureterectomy (SU) has recently been attracting attention as a treatment strategy for ureteral cancer. Hung et al. examined 112 patients with pure UTCC treated surgically and concluded that the oncological outcomes of SU were not inferior to those of RNU for ureter cancer [8]. These findings imply that a sufficiently effective treatment for localized ureter cancer is not only RNU with excision of the bladder cuff, but also topical treatments including SBRT.

No acute or late adverse events were observed in any of the three cases presented herein. However, a longer observation period may be needed to more accurately estimate late adverse events.

In conclusion, SBRT may become one of the treatment choices for inoperable localized UTCC. Further studies including a larger number of cases and longer observation periods are needed.

## Figures and Tables

**Table 1:** Summary of case presentations.

	Case 1	Case 2	Case 3
Age	87	87	85
Sex	F	M	F
Tumor location in ureter	Orifice	Distal one-third	Distal end
TNM staging	T2N0M0	T2N0M0	T2N0M0
Cause for avoidance of surgery	CKD Rheumatism	Patient refusal	Patient refusal
Dose/fractionations	60 Gy/10 fr	50 Gy/10 fr	60 Gy/10 fr
Tumor size in 3 months	Decreased	Decreased	Decreased
Acute adverse events	None	None	None
LPFS (month)	12	12	24
Recurrence of disease	None	None	Bladder
OS (month)	33	13	38
Cause of death	Rheumatism	Bacterial pneumonia	Cancer

Abbreviations: CKD, Chronic kidney disease; LPFS, Local progression-free survival; OS, Overall survival.

## References

[1] Ito Y., Kikuchi E., Tanaka N. (2011). Preoperative hydronephrosis grade independently predicts worse pathological outcomes in patients undergoing nephroureterectomy for upper tract urothelial carcinoma. *The Journal of Urology*.

[2] Rouprêt M., Zigeuner R., Palou J. (2012). European guidelines for the diagnosis and management of upper urinary tract urothelial cell carcinomas: 2011 update. *Actas Urologicas Espanolas*.

[3] Cozad S. C., Smalley S. R., Austenfeld M., Noble M., Jennings S., Reymond R. (1992). Adjuvant radiotherapy in high stage transitional cell carcinoma of the renal pelvis and ureter. *International Journal of Radiation Oncology, Biology, Physics*.

[4] Santacaterina A., Platania A., Palazzolo C. (2015). Very elderly (>80 years), frail patients with muscle-invasive bladder cancer and comorbidities: is curative irradiation feasible?. *Tumori Journal*.

[5] Onishi H., Araki T., Shirato H. (2004). Stereotactic hypofractionated high-dose irradiation for stage I nonsmall cell lung carcinoma: clinical outcomes in 245 subjects in a Japanese multiinstitutional study. *Cancer*.

[6] Pontoriero A., Iatì G., Mondello S. (2015). High-dose robotic stereotactic body radiotherapy in the treatment of patients with prostate cancer: preliminary results in 26 patients. *Technology in Cancer Research & Treatment*.

[7] Hall M. C., Womack S., Sagalowsky A. I., Carmody T., Erickstad M. D., Roehrborn C. G. (1998). Prognostic factors, recurrence, and survival in transitional cell carcinoma of the upper urinary tract: a 30-year experience in 252 patients. *Urology*.

[8] Hung S. Y., Yang W. C., Luo H. L., Hsu C.-C., Chen Y. T., Chuang Y. C. (2014). Segmental ureterectomy does not compromise the oncologic outcome compared with nephroureterectomy for pure ureter cancer. *International Urology and Nephrology*.

